# Basal and IL-1β enhanced chondrocyte chemotactic activity on monocytes are co-dependent on both IKKα and IKKβ NF-κB activating kinases

**DOI:** 10.1038/s41598-021-01063-2

**Published:** 2021-11-04

**Authors:** Eleonora Olivotto, Manuela Minguzzi, Stefania D’Adamo, Annalisa Astolfi, Spartaco Santi, Mariagrazia Uguccioni, Kenneth B. Marcu, Rosa Maria Borzì

**Affiliations:** 1 Laboratorio RAMSES, IRCCS Istituto Ortopedico Rizzoli, Bologna, Italy; 2 Dipartimento di Scienze Mediche e Chirurgiche (DIMEC), Università di Bologna, Bologna, Italy; 3 Department of Translational Medicine, University of Ferrara, Ferrara, Italy; 4 Institute of Molecular Genetics “Luigi Luca Cavalli-Sforza”, Unit of Bologna, CNR, Bologna, Italy; 5 IRCCS, Istituto Ortopedico Rizzoli, Bologna, Italy; 6 Institute for Research in Biomedicine, Università della Svizzera Italiana, Bellinzona, Switzerland; 7 Department of Biomedical Sciences, Humanitas University, Pieve Emanuele, Milan Italy; 8 Departments of Biochemistry and Cell Biology and Pathology, SUNY, Stony Brook, NY USA; 9 Laboratorio di Immunoreumatologia e Rigenerazione Tissutale, IRCCS Istituto Ortopedico Rizzoli, Bologna, Italy

**Keywords:** Cell biology, Molecular biology, Rheumatology

## Abstract

IKKα and IKKβ are essential kinases for activating NF-κB transcription factors that regulate cellular differentiation and inflammation. By virtue of their small size, chemokines support the crosstalk between cartilage and other joint compartments and contribute to immune cell chemotaxis in osteoarthritis (OA). Here we employed shRNA retroviruses to stably and efficiently ablate the expression of each IKK in primary OA chondrocytes to determine their individual contributions for monocyte chemotaxis in response to chondrocyte conditioned media. Both IKKα and IKKβ KDs blunted both the monocyte chemotactic potential and the protein levels of CCL2/MCP-1, the chemokine with the highest concentration and the strongest association with monocyte chemotaxis. These findings were mirrored by gene expression analysis indicating that the lowest levels of CCL2/MCP-1 and other monocyte-active chemokines were in IKKαKD cells under both basal and IL-1β stimulated conditions. We find that in their response to IL-1β stimulation IKKαKD primary OA chondrocytes have reduced levels of phosphorylated NFkappaB p65pSer536 and H3pSer10. Confocal microscopy analysis revealed co-localized p65 and H3pSer10 nuclear signals in agreement with our findings that IKKαKD effectively blunts their basal level and IL-1β dependent increases. Our results suggest that IKKα could be a novel OA disease target.

## Introduction

Osteoarthritis (OA) is the most common joint disorder and the major cause of disability in the adult population^1^. It is now well established that OA is not only the result of loss of cartilage homeostasis but a whole-joint disorder involving all the joint tissues^2^.

Historically defined as a non-inflammatory disease, OA is now considered a condition involving persistent low-grade inflammation, oxidative stress^3^ and activation of innate inflammatory pathways with the recruitment of monocytes, lymphocytes, and other leukocytes in the synovial tissue^4^. This condition contributes to perpetuate and enhance the pathophysiology of the disease^5^.

Exploiting surgical animal models, the slow progressive human OA disease has been recapitulated^6^, thus allowing to dissect some key target genes by functional genomics^7^: interleukin-1β (IL-1β) and a disintegrin and metalloproteinase with thrombospondin motifs 5 (ADAMTS5). IL-1β has been therefore widely used in vitro to reproduce the inflammatory-catabolic environment of the disease with multiple crosstalk among the different cell types involved as recently reviewed^8^. The inflammatory response is characterized by coordinated activation of various signalling pathways that regulate expression of mediators, among them nuclear factor kappa-light-chain-enhancer of activated B cells (NF-κB) is reported to play a prominent role in OA^8,9^ both in cartilage and synovial tissue^10^.

Following inflammatory stimuli, NF-κB heterodimers are activated in the cytoplasm by the site specific amino-terminal phosphorylation of NF-kappa-B inhibitor alpha (IκBα) by the I-kappa-B kinase (IKK) signalosome complex that targets it for proteosomal destruction resulting in NF-κB nuclear translocation to induce gene transcription. The IKK complex consists of two serine-threonine kinases, IKKα and IKKβ, and NF-kappa-B essential modulator (NEMO, also called IKKγ), a regulatory or docking protein. IKKβ is the dominant IκBα kinase in vivo, whose activation is essential for the nuclear translocation of canonical NF-κB heterodimers (including p65(RelA):p50 and cRel:p50). In contrast, IKKα only occasionally acts as the IκBα kinase but instead has been reported to play this role in cells with IKKβ inhibition^11^. Noteworthy, IKKα may also act as a nucleosomal kinase to activate transcription of some canonical NF-κB targets^12,13^. Unlike IKKβ, IKKα is uniquely required in vivo for the activation of the non-canonical or alternate NF-κB pathway, which is delayed and requires proteolytic processing of key intermediates^9^.

IKKα and IKKβ are essential kinases for activating NF-κB transcription factors that regulate not only inflammation but also cellular differentiation. OA is an age-related disease that mainly affects cartilage that exhibits loss of correct differentiation^14^ driven by inflammatory loops. Interplay of inflammation, oxidative stress, senescence and altered differentiation sustains OA pathogenesis^3^ and IKK differential roles in supporting OA pathogenesis emerged in the last years^9,15^. Specifically, IKKα has been recognized to exert a prevalent role in the loss of maturational arrest of chondrocytes and their progression towards hypertrophy and terminal differentiation^15^, that is also sustained by activation of latent proteolysis^16^.

By virtue of their small size enabling penetration into the complex network of cartilage extracellular matrix, chemokines mediate crosstalk between OA cartilage chondrocytes with other joint compartments and contribute to immune cell chemotaxis to the synovium and the synovial space^17^. Recruitment of monocytes to the synovium has been evidenced as a pivotal event in driving synovial inflammation and linking innate immunity to OA^18,19^. In the surgical destabilization of the medial meniscus (DMM) mouse model of OA, C–C motif chemokine 2 (CCL2)/Monocyte chemoattractant protein 1 (MCP-1) is among the earliest induced genes, in a matter of hours after DMM^20^, and mostly involved in pain rather than in cartilage degradation.

Since many chemokines are known to be direct NF-κB targets^21^, here we employed retroviral mediated transduction of short hairpin RNA (shRNA) to stably and effectively ablate the expression of each IKK^15^ thus determining their individual contributions for monocyte chemotaxis in response to chondrocyte conditioned media. This additional information would be useful in developing strategies to target the upstream NF-κB signaling events, in order to blunt inflammation, chemokine release and monocyte activation and recruitment. The effects of the IKK knockdowns (KDs) in chondrocytes were evaluated in both monolayer and differentiated micromass cultures.

## Materials and methods

### Isolation of primary osteoarthritic chondrocytes and retroviral transduction

Knee cartilage samples were collected from OA patients undergoing knee arthroplasty. The study was carried out in compliance with the Helsinki declaration, and approved by the IRCCS Istituto Ortopedico Rizzoli ethical committee (protocol “OA-TARGET”, Prot.gen.n.ro 0009882), including documentation of informed written patient consent. Processing of articular cartilage and experimental procedures described below were performed in accordance with the relevant guidelines and regulations.

After tissue retrieval, all patient identifiers were removed, and samples were coded by arbitrary designations to distinguish them solely for experimental purposes. Primary chondrocytes were isolated by mean of sequential enzymatic digestion from cartilage of OA patients (n = 7), as described in^22^. In some cases, before finely mincing the cartilage for establishing chondrocyte primary cultures, full thickness cylinders of cartilage comprising all the cartilage layers from superficial to deep cells were obtained by punching with a biopsy needle kept perpendicular to the articular surface, as described in^23^. The tissues were embedded in O.C.T. compound (Tissue-Tek), stored at − 80 °C, sectioned and processed for immunohistochemistry, as described below.

OA chondrocytes, expanded in vitro until confluence, were then seeded at low density and transduced by spinoculation with amphotyped retroviruses prepared from Phoenix A packaging cells (provided by Dr. Gary Nolan at Stanford University)^15^. As previously detailed, cells were used at passage 1 (p1) to avoid the loss of the correct chondrocyte phenotype, in agreement with published guidelines^24^. Knockdowns (KDs) of IKKα and IKKβ were achieved with retroviral vectors containing IKKα- or IKKβ-specific shRNA (shOligos) subcloned into the pSuper.retro (Puro) moloney retroviral vector. The phenotypes of chondrocytes stably transduced with IKKα or IKKβ specific shRNAs were compared with that of a negative control (GL2), corresponding to cells obtained from the same patient but infected by a retroviral vector harbouring a firefly luciferase-specific shRNA^15^. Efficiency of the knockdown was assessed by western blot^15^.

### Chondrocytes culture condition

After KD validation^15^, stably transduced chondrocytes were seeded at “high density” either in monolayers (2-D) or into differentiating micromass cultures (3-D) as previously described^15,23^.

Some chondrocytes were seeded into micromasses, and left for 1 week. Other chondrocytes were seeded in monolayer but at high density (100,000/cm^2^), a condition that helps in recovering and maintaining the correct phenotype^25,26^, which corresponds to 400,000 cells/well in 12‐well culture plates for collection of supernatants and immunoblotting experiments and 120,000 cells in 8‐well chamber slides for immunofluorescence experiments in Dulbecco's Modified Eagle Medium supplemented with 1% penicillin/streptomycin and 10% fetal bovine serum (FBS). After 5 days the medium was replaced with fresh serum‐free culture medium and cells were exposed to 2 ng/ml IL‐1β to reproduce the inflammatory environment of OA cartilage^8^. This OA proinflammatory stimulus was maintained for different time lengths, according to the different type of functional response under investigation. A long term 24 h (hr) stimulation was used for the evaluation of the differential chemokine repertoire in the supernatant of cultures with different IKK phenotype, which was also collected to establish chemotaxis experiments. A short term (8 h) stimulation was used to undertake investigation of gene expression changes. Finally, a kinetic (0, 30, 60 and 120 min) IL-1β exposure was used to explore the differential signalling by both western blot and immunofluorescence. Unstimulated cells were used as control.

### Isolation of primary peripheral blood mononuclear cells

Blood from healthy donors (HD) was provided as buffy-coats by the Central Laboratory of Swiss Red Cross (Basel, Switzerland), and usage approved by the Cantonal Ethics Committee of Ticino, Switzerland (TI-2018-02166). Informed consent was obtained from all subjects. Collection of blood samples from healthy donors and isolation of monocytes were performed in accordance with the relevant guidelines and regulations. All samples were processed within 24 h after blood withdrawal. Peripheral blood mononuclear cells (PBMCs) were isolated using Ficoll-Hypaque density centrifugation. Monocytes, CD14+, were isolated by positive immunoselection procedure (130-050-201, CD14 MicroBeads, Miltenyi Biotec, Germany) according to the manufacturer’s instructions.

### Chemotaxis assay

Chemotaxis of monocytes towards chondrocyte conditioned media was assayed in 48-well Boyden microchambers (Neuro Probe Inc., Cabin John, MD) by using polyvinylpryrrolidone-free polycarbonate membranes with 5 µm pores (Nucleopore). Supernatants (7 different experiments) diluted in chemotaxis buffer (RPMI 1640, 20 mM Hepes, pH 7.4, containing 0.05% pasteurized plasma protein from the Swiss Red Cross Laboratory, Bern, Switzerland) were placed in the lower wells. In all inhibition experiments, CCL26/Eotaxin-3 was placed in the upper and lower wells. 5 × 10^4^ monocytes/well were resuspended in chemotaxis buffer. After 90 min of incubation the membrane was removed, washed on the upper side with phosphate buffered saline, fixed, and stained. Migrated cells were counted at 1000 magnification in five fields/well. All experiments were performed in triplicates.

### Oligo GEarrays (SuperArray)

To obtain an integrated overview of the effects of IL-1β stimulation on the mRNA expression of chemokines and chemokine receptors in chondrocytes with different phenotypes these correlated genes were explored by mean of the Oligo GEarrays (SuperArray).

Total RNA from stably transduced chondrocytes cultured as detailed above in control and IL-1β stimulated conditions for 8 h, was extracted using TRIZOL reagent (Invitrogen) following manufacturer’s instructions and the DNA contamination was removed by digestion with RNase-free DNase (Ambion) as described in Olivotto et al.^22^.

Structural integrity, yield and purity of RNA were determined photometrically (NANODROP 2720, Thermal Cycler, Applied Biosystem) and by agarose gel electrophoresis. Total RNA was amplified (cRNA) and labelled with biotinylated-UTP using the TrueLabeling-AMP 2.0 kit (SuperArray). The cRNA was used for hybridization to the Oligo GEArray microarray human chemokines and receptors microarray (OHS-022, SuperArray) according to the manufacturer’s protocol. The final detection step exploited the use of alkaline phosphatase-conjugated streptavidin and the chemiluminescent substrate CPD-Star. The microarray contains 128 oligonucleotides probes printed on a nylon membrane with a non-contact printing technology in row of 8 columns. The membrane contains 4 different housekeeping genes and each gene analyzed is represented in quadruplicate.

Differential gene expression levels in untreated and 2 ng/ml IL-1β treated chondrocytes was revealed by chemiluminescence signals. The intensity of each spot of each quadruplicate was quantified with Carestream Molecular Imaging Software 5.0 (Carestream Health, New Haven, CT).

### Real time RT-PCR

Up to to 2.5 µg of RNA extracted from GL2, IKKαKD and IKKβKD chondrocytes in basal or IL-1β stimulated (8 hours) conditions were reverse transcribed with the Superscript VILO cDNA Synthesis kit (Life Technologies) according to the manufacturer’s instructions. CCL2/MCP-1 messenger RNA (mRNA) was quantified by real-time reverse transcriptase (RT) polymerase chain reaction (PCR) using SYBR Green PCR kit (Quiagen) in a Light Cycler 2.0 instrument (Roche Diagnostics), with values normalized to the expression of GAPDH mRNA^27^. Annealing temperature was 56 °C for both GAPDH primers (forward: CGGAGTCAACGGATTTGG; reverse: CCTGGAAGATGGTGATGG) and CCL2/MCP-1 primers (forward: GAAGCTCGCACTCTCGCCT; reverse: GAGTGTTCAAGTCTTCGGA).

### Immunoblotting

Stable transduced chondrocytes in both untreated and IL-1β treated conditions were processed for western blot analysis, exploiting SNAP-ID 2.0 device (Merck-Millipore) essentially as described in Minguzzi et al.^28^, loading the same cell equivalents for each lane (150,000 or 200,000 cells, according to the different experiment). Primary antibodies against the antigens were as follows: IKKα (mouse monoclonal, clone B78-1, used at 0.5 µg/ml, BD Pharmingen # 556532), IKKβ (polyclonal rabbit anti-human IKKβ, used 1:1000, Cell Signaling Technology, #2684), Phospho-NF-κB p65(Ser536) (rabbit monoclonal antibody, clone 93H1, used at 1:1000, Cell Signaling Technology #3033), phospho-histone H3 (Ser10) (mouse monoclonal, clone CMA312, used at 0.6 µg/ml, MERCK # 05-1336). β-actin (mouse monoclonal, clone AC-74, used at 0.8 µg/ml Sigma # A2228) served as loading control. Appropriate anti-species HRP conjugated secondary antibodies were from Jackson ImmunoResearch Laboratories.

### Immunohistological and immunofluorescence analysis

To investigate the distribution of CCL2/MCP-1 expressing chondrocytes across full thickness cartilage, CCL2/MCP-1 immunohistochemistry (IHC) was performed on 5 μm sections prepared from OA human cartilage explants. The latter were obtained by punching perpendicularly the articular cartilage with a biopsy needle, in order to obtain tissue cylinders containing all of the cartilage layers. The cylinders were immediately embedded in OCT (Tissue-Tek, Sakura, USA), snap frozen in liquid nitrogen (LN2) and stored at − 80 °C until the time of processing. Then, 5 µm sections were obtained and fixed with 4% paraformaldehyde (PFA) for 30 min and processed for CCL2/MCP-1 IHC, according to the Streptavidin Super Sensitive IHC Detection Systems protocol (Biogenex, San Ramon, CA). Before immunodetection, the sections were treated for antigen unmasking with a solution of 0.02 U/ml Chondroitinase ABC (SIGMA) in 50 mM Tris/HCl pH 8.0 for 20 min at 37 °C. Then, blocking of non-specific signals was carried out with tris-buffered saline (TBS) with the addition of 5% normal goat serum (NGS), 2% bovine serum albumin (BSA) and 0.1% Triton-100. Primary antibodies, diluted in TBS + 3% NGS + 2% FBS + 0.1% NaAz + 0.1%Triton were anti-MCP-1 mouse monoclonal antibody, IgG1, Pharmingen, Cat 20520D, used at 1 μg/ml along with mouse IgG1 isotype control, R&D at the same concentration. Signals were developed with the SuperSensitive IHC and with fast red substrate. Nuclei were counterstained with haematoxylin and slides mounted with Aqua-Mount Mounting Medium (Thermo Scientific). Images were captured with a Nikon Eclipse 90i microscope equipped with NIS (Nikon Imaging Software) elements (Nikon Inc).

Other tissue sections obtained from cartilage explants were used to evaluate the differential IKKα expression in areas with conserved (Non-OA) versus areas with marked perturbation of cartilage extracellular matrix (OA), as assessed by Safranin-O staining. The sections were treated as detailed above, but the following primary antibody was used: anti IKKalpha Mouse monoclonal antibody, IgG2b, Pharmingen, Cat 556532 used at 5 µg/ml along with Mouse IgG2b Isotype control.

Double immunofluorescence (IF) was performed to investigate the differential pattern of major signalling events. IF was performed essentially as described in Pagani et al.^27^ on stable transduced chondrocyte cultured in 8‐well chamber slides untreated (0 min) or IL-1β treated (30, 60, 120 min). The experiment was carried out to correlate the intensity and spatial occurrence of the nuclear translocation of p65 with the phosphorylation of serine 10 in Histone H3 (H3pSer10).

At the end of IL-1β stimulation, the cells were fixed with 4% PFA for 30 min, followed by a brief exposure to 90% methanol for 5 min on ice, in order to increase access to nuclear content. Then, the IF procedure was carried out with 5 min TBS washes between the steps. Primary and secondary antibodies were delivered diluted in TBS with the addition of 3% BSA and 0.1% Triton-X-100.

Before primary antibody incubation, the samples were pre-treated for antigen unmasking with 0.02 U/ml Chondroitinase ABC (SIGMA) in 50 mM Tris/HCl pH 8.0 for 20 min at 37 °C and permeabilized with 0.2% Triton in TBS solution for 5 min at room temperature (RT). After extensive washes with TBS, the non-specific bindings were blocked with 5% BSA, and 0.1% Triton in TBS for 30 min at RT and washed again.

Then, the NF-κB p65 staining was performed with 5 μg/ml of rabbit Anti-NF-κB p65 antibody (ChIP Grade, ABCAM #ab7970) and the phospho Histone H3 staining with 5 µg/ml of phospho-histone H3 (Ser10) (mouse monoclonal, clone CMA312, MERCK** #** 05-1336**)** incubated overnight at 4 °C. Following extensive washing, the signals were revealed with the secondary antibodies: 15 µg/ml donkey anti-Rabbit IgG (H + L) Alexa Fluor 488 conjugate (Novex A31572) and 15 µg/ml donkey anti-Mouse IgG (H + L) Alexa Fluor 555 conjugate (Novex A31570), respectively. Nuclei were counterstained with 5 µg/ml Hoechst 33342 (Sigma) and slides were mounted with the addition of an anti-fading reagent (1% 1,4 Diazabicyclo (2.2.2) Octane (DABCO, SIGMA) in 90% glycerol in 0.1 M pH 8.0 Tris–HCl), sealed with nail polish and stored refrigerated in the dark for subsequent analysis.

Images were taken at high magnification with a NIKON A1-R confocal laser scanning microscope equipped with a NIKON 20×, 0.95 NA objective lens, and with 405, 488 and 561 nm laser lines to excite Hoechst 33342 (blue), Alexa Fluor 488 (green) and Alexa Fluor 555 (red) fluorescence signals, respectively. Emission signals were detected by a photomultiplier tube (DU4) preceded by emission filters BP 525/50 nm and BP 595/50 nm for Alexa Fluor 488 and Alexa Fluor 555, respectively^29^. Laser scanning, image acquisition and processing were performed with NIKON Imaging Software NIS Elements AR-4 (NIKON Inc., USA). Optical sections were spaced *0.5 μm along the z axis and were digitized with a scanning mode format of 1024 × 1024 pixels and 4096 grey levels.

To account for the variability of cell behavior a large image was obtained merging from four to five different fields each acquired with a 20× objective in order to collect information from the entire well. For image purposes, each field was further divided in 3 × 3 areas, and a representative image was presented in Fig. 5. A further 3× magnification was also shown (bottom row).

Co-localization analysis, as described below was undertaken for 26 different areas randomly distributed in each field.

The double immunofluorescence was carried out for all the times (0, 30, 60 and 120 min) of IL-1β stimulation, but a deeper co-localization analysis was undertaken for the 60 min time point. Based on previous findings^30^, the combined p65/H3pSer10 picture obtained at this time point likely reflects downstream transcription of RelA target genes that also depend on H3 phosphorylation for NF-κB recruitment.

The co-localization of the fluorochromes was evaluated using Pearson’s correlation coefficient^31^, that provides information about the similarity of shape between images and does not take into account image intensity. It is a value computed to be between –1 and 1 and was calculated on the representative images by using NIS element software (Nikon Instruments Europe B.V., Nikon, Amsterdam, The Netherlands).

The Pearson’s correlation coefficient was also expressed as increased value after 1 h of treatment compared to the basal condition.

### Multiplex assessment of chemokine levels in supernatants

The major chemokines active on monocytes (CCL2/MCP‐1, CCL3/MIP-1α, CCL4/MIP-1β, CCL5/RANTES) were simultaneously evaluated in culture supernatants by means of commercially available multiplex bead‐based sandwich immunoassay kits with high sensitivity and dynamic range (Bio‐Plex Protein Array System, Bio‐Rad Laboratories, Hercules, CA) coupled with Bio-Plex Pro Human Cytokine MCP-1 (MCAF), RANTES, MIP-1beta and MIP-1alpha Sets.

Data were analyzed by using the Bio‐Plex Manager software version 6.0 (Bio‐Rad Laboratories Hercules, CA). Standard levels between 70 and 130% of the expected values were considered accurate. A value half of the lower limit of quantification (LLQ) was assigned to analytic concentrations less than the LLQ, while a value equal to the upper limit of quantification (ULQ) was assigned to analytic concentrations over the ULQ.

In general, at least six standards were accepted and used to establish standard curves following a Five-Parameter Logistic (5-PL) regression model. Sample concentrations were immediately interpolated from the standard curves.

### Statistical analysis

The graphs represent the cumulative analysis of at least three different experiments performed with cells obtained from likewise different patients, or otherwise stated in the Figure legend. All the data presented in graphs are expressed as means ± standard deviation (SD). Means of groups were compared with GraphPad Prism 5 statistical software (GraphPad Software, Inc. La Jolla, CA, USA) and analyzed for statistical significance (* = p < 0.05, ** = p < 0.01, *** = p < 0.001). Differences were considered statistically significant at p < 0.05. Comparison of different group of samples was performed by mean of the Student’s t-test (two-tailed), or ANOVA (with Tukey’s post hoc test) either for paired or unpaired samples, where appropriate. Correlations were calculated with Pearson r, p value two-tailed.

## Results

### Retroviral mediated IKK KD yielded penetrant and stable KD of each IKK

For each functional study listed below, a comparison was undertaken between the wild type IKK expressing control (GL2) and each IKKα and β KD, in basal or IL-1β stimulated conditions. The primary cultures used in the study showed penetrant and stable KD of each IKK as previously shown^15,16,23,32^ and summarized in Supplementary Fig. 1.

### Chemokine gene expression in chondrocytes indicates that IKKαKD markedly affects both basal and IL-1β-induced levels

**Figure 1:**
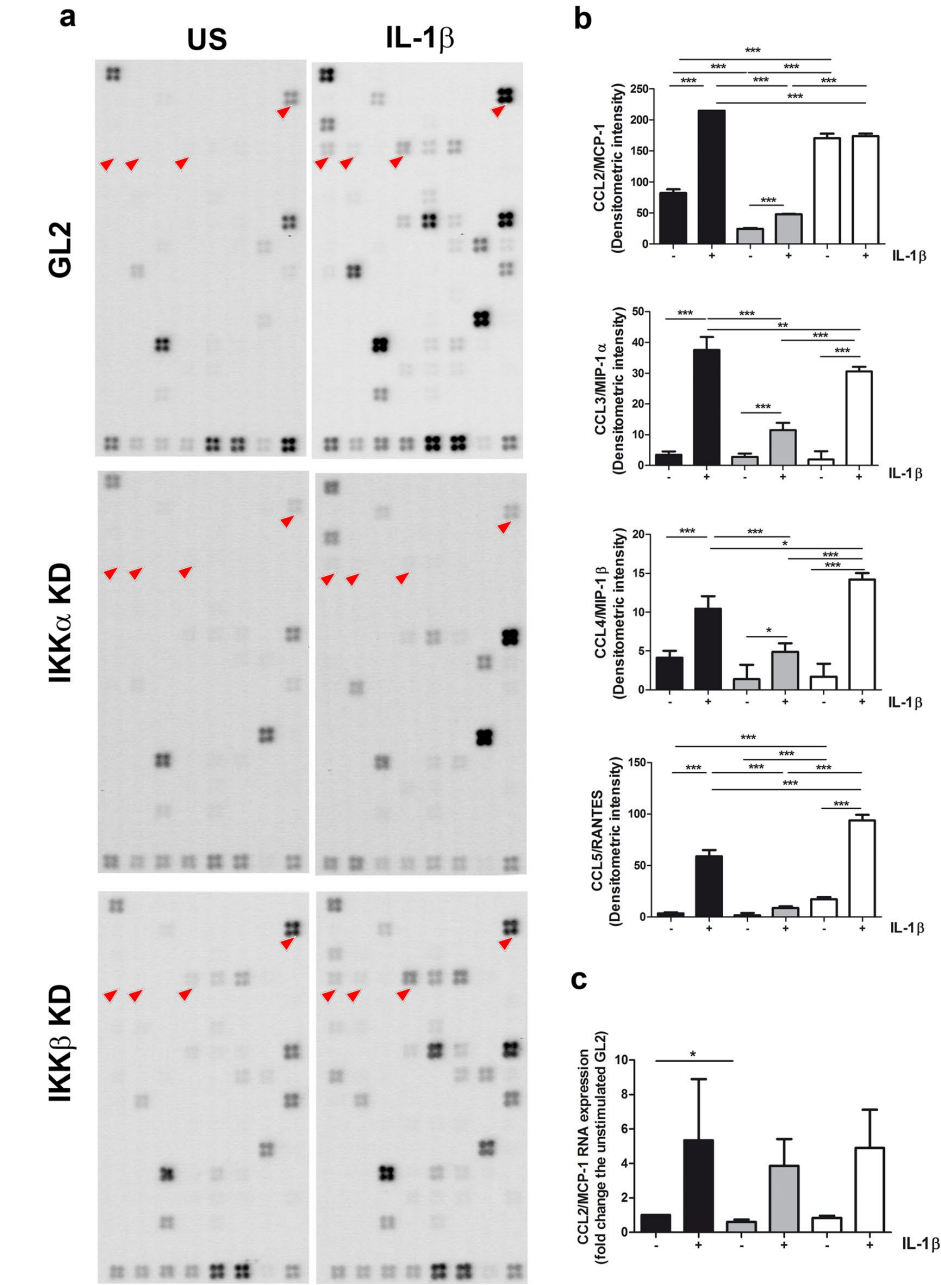
IKK dependency of the transcription of monocyte recruiting chemokines. (**a**) Total RNA obtained from GL2, IKKαKD or IKKβKD chondrocytes in basal conditions or following 8 h exposure to 2 ng/ml IL-1β was probed against the Oligo GEArray microarray human chemokines and receptors microarray (OHS-022, SuperArray). The array layout is indicated in Supplementary Fig. 2. The probe positions for the monocyte active chemokines (CCL2/MCP-1, CCL3/MIP-1α, CCL4/MIP-1β and CCL5/RANTES) are indicated by red arrows. (**b**) For each chemokine, every single dot signal of the quadruplicate was quantified by densitometric measurements, and signals were compared across the different conditions: GL2 (black pattern), IKKαKD (gray pattern) or IKKβKD (white pattern) chondrocytes in control (−) or IL-1β stimulation (8 h, +). The different Y axis scale shows the different expression magnitudes of these chemokines in the three different genotypes. Cumulative results of the four replicates are shown as mean ± SD. Chemokine gene expression levels in both basal and IL-1β stimulated conditions were compared across the different phenotypes by ANOVA, followed by Tukey’s post hoc test. The differences were considered significant when p < 0.05 with: **p < 0.01; and ***p < 0.001. (**c**) Real Time PCR performed with RNA obtained from cultures derived from three different patients confirms an evident IL-1β induction and in basal conditions lower CCL2/MCP-1 RNA in IKKαKD compared to the GL2 controls.

To further explore the IKK dependence of major CC chemokines after IL-1β stimulation, we performed an Oligo GEArray microarray designed to profile the expression of a focused gene panel from a specific biological pathway or disease state. We analysed at the same time the IL-1β dependent increased expression of 128 genes corresponding to chemokines and their receptors in one patient. Figure 1a shows probes spotted in quadruplicate, as detailed in the array layout shown in Supplementary Fig. 2. We focused our attention to the four chemokines active on monocytes also investigated in the Bioplex assay (evidenced by red arrows in the membranes of Fig. 1a that shows the microarray results for un-stimulated (US), left and IL-1β stimulated conditions (right). For each chemokine, every single dot signal of the quadruplicate was quantified, and signals were compared across the different conditions (IKK proficient or deficient cells and in basal or IL-1β stimulated conditions) by ANOVA (Fig. 1b). Some differences were already evident under basal conditions. CCL2/MCP-1 levels were significantly lower in IKKαKD cells compared to both GL2 and IKKβKD cells. On the other hand, CCL5/RANTES was significantly higher in IKKβKD cells compared to both GL2 and IKKαKD cells.

In GL2 samples, IL-1β stimulation led to a marked increase in gene expression for all of the chemokines. In IKKαKD samples, the gene expression levels of the four chemokines were very low and close to the limits of detection both at basal levels and after inflammatory stimulation. IKKαKD samples also showed a modest, yet significantly increased chemokine expression after inflammatory stimulation compared to the basal condition for CCL2/MCP-1 and CCL3/MIP-1α. The increase was more modest for CCL4/MIP-1β, while CCL5/RANTES was almost unaffected by IL-1β stimulation.

Otherwise, IKKβKD samples presented high gene expression levels for CCL2/MCP-1 both at basal levels and after IL-1β stimulation, which appeared to be almost ineffective in inducing further transcription. In IKKβKD compared to GL2 samples, IL-1β induction was significantly higher for CCL4/MIP-1β and CCL5/RANTES, and significantly lower for CCL3/MIP-1α.

As detailed in the figures, for both the IKK KD samples, the differences compared to the GL2 samples were statistically significant after IL-1β stimulation. In all cases, IKKαKD showed markedly reduced IL-1β dependent chemokine induction in comparison with both the GL2 control and IKKβKD phenotype. Interestingly, compared to GL2, IKKβKD cells showed statistically significant lower CCL2/MCP-1 and CCL3/MIP-1α levels, and higher for CCL4/MIP-1β and CCL5/RANTES.

The difference in basal CCL2/MCP-1 gene expression in the three phenotypes, and particularly the reduced CCL2/MCP-1 expression in IKKαKD, was also confirmed by real time PCR (Fig. 1c, showing results obtained with chondrocytes from three different patients in basal and IL-1 stimulated conditions). We confirmed that CCL2/MCP-1 gene expression was detected at basal level in monolayer cultures of chondrocytes with the three different phenotypes. The expression was higher in the wild type (GL2) compared to the IKKαKD samples (p = 0.0393, n = 3).

### Chemokine protein expression in conditioned media from chondrocytes with either IKKα or IKKβ knockdown showed that CCL2/MCP-1 plays a major role in monocyte chemotaxis

**Figure 2:**
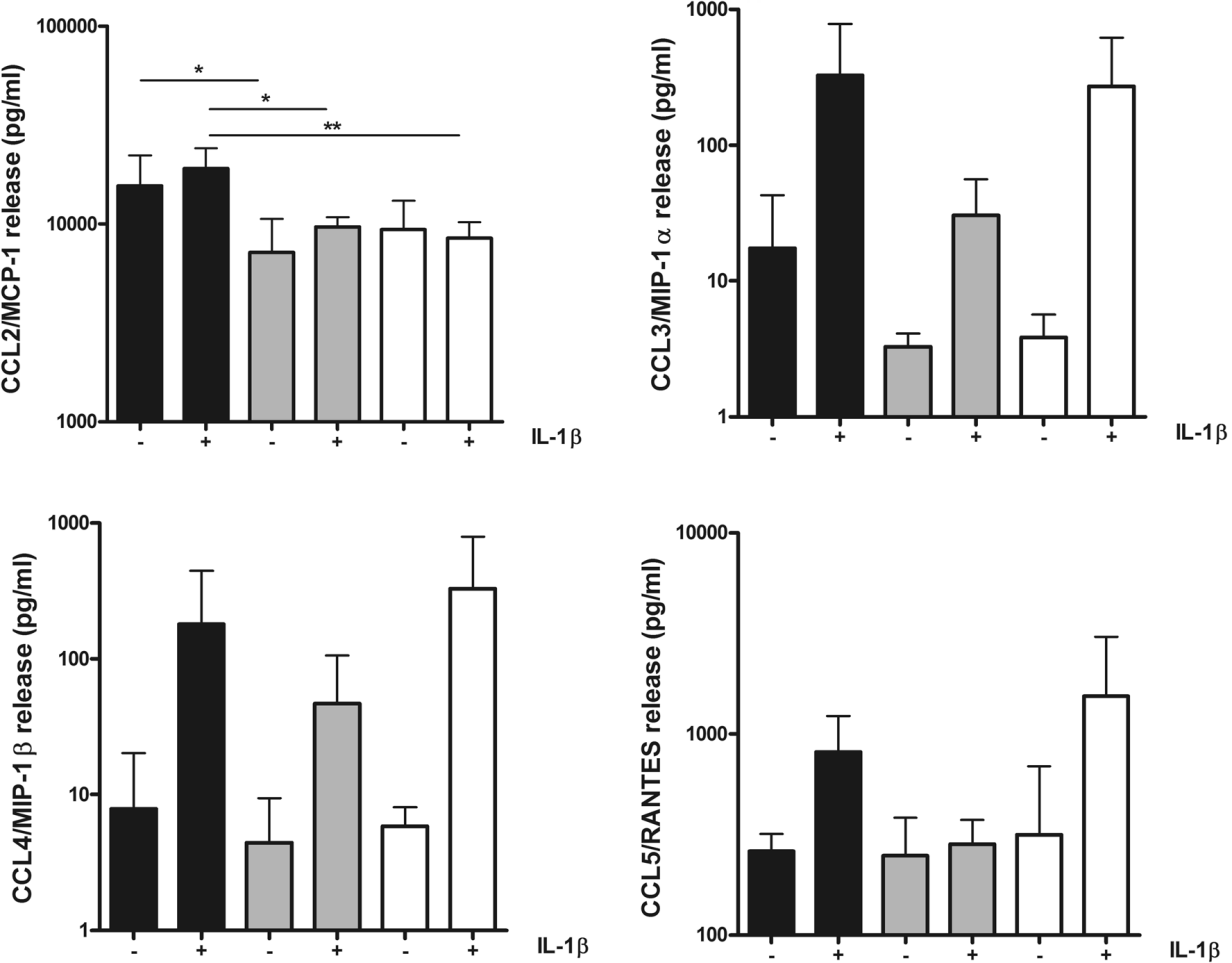
IKK dependency of the release of monocyte recruiting chemokines. Supernatants obtained from high density cultures of GL2 (black pattern), IKKαKD (gray pattern) or IKKβKD (white pattern) chondrocytes in control condition (−) or after IL-1β stimulation (24 h, +) were collected and assessed for the level of CCL2/MCP-1, CCL3/MIP-1α, CCL4/MIP-1β and CCL5/RANTES. Data are reported as mean ± SD (n = 3 different experiments each performed with cells of different patients). The means of the groups were compared by ANOVA, followed by Tukey’s post hoc test. The differences were considered significant when p < 0.05 with *p < 0.05; **p < 0.01.

To further investigate the role of the monocyte-active chemokines under study, their protein levels were multiplex-assessed by a fluorescence bead-based assay endowed with a high sensitivity and dynamic range (BIO-RAD Bioplex). Data were obtained from the conditioned media of chondrocyte monolayers with either IKKα or IKKβ KD and control GL2 phenotype with or without IL-1β stimulation for 24 h (Fig. 2, n = 3).

Each of the four chemokines were analyzed, and in agreement with the Oligo GEArray microarray data, CCL2/MCP-1 overwhelmed the levels of the other chemokines. Indeed, considering its basal level in GL2 chondrocytes, CCL2/MCP-1 was expressed at nearly 60-fold higher levels than CCL5/RANTES, nearly 900-fold greater than CCL3/MIP-1α and nearly 2000-fold above CCL4/MIP-1β.

CCL2/MCP-1 protein expression was high in all samples already under un-stimulated conditions, particularly in GL2 supernatants (mean ± SD: 15,594 ± 6612). Noteworthy, significantly decreased basal levels of CCL2/MCP-1 were found in IKKαKD cells compared to GL2, while after IL-1β stimulation, a significantly lower induction was found both in IKKαKD cells (*) and IKKβKD cells (**) (Fig. 2).

Under un-stimulated conditions, the protein level of both CCL3/MIP-1α and CCL4/MIP-1β was very low for each of the three phenotypes (GL2, IKKαKD and IKKβKD). After IL-1β stimulation, chemokine release was markedly although not significantly increased in both GL2 and IKKβKD samples, while the increase in IKKαKD samples was extremely low (Fig. 2).

Similar to CCL3/MIP-1α and CCL4/MIP-1β, basal expression of CCL5/RANTES did not change across the three phenotypes, but IL-1β stimulation strongly increased its release in GL2 and IKKβKD supernatants, while was almost ineffective in IKKαKD samples (Fig. 2).

Therefore, CCL2/MCP-1 production following IL-1β was co-dependent on IKKα and IKKβ, while stimulus induced expression of CCL3/MIP-1α, CCL4/MIP-1β and CCL5/RANTES appeared to be largely dependent on IKKα and not IKKβ.

### IKKα and IKKβ knockdown blunted the monocyte chemotactic potential of chondrocyte conditioned media

The chemotactic activity of conditioned media from OA chondrocytes was tested using primary human monocytes. To fully dissect the contribution of each of the two major signalosome kinases in both control and IL-1β treated conditions, supernatants were collected from human primary chondrocytes stably transduced with either IKKαKD or IKKβKD or control (GL2) shRNA.

**Figure 3:**
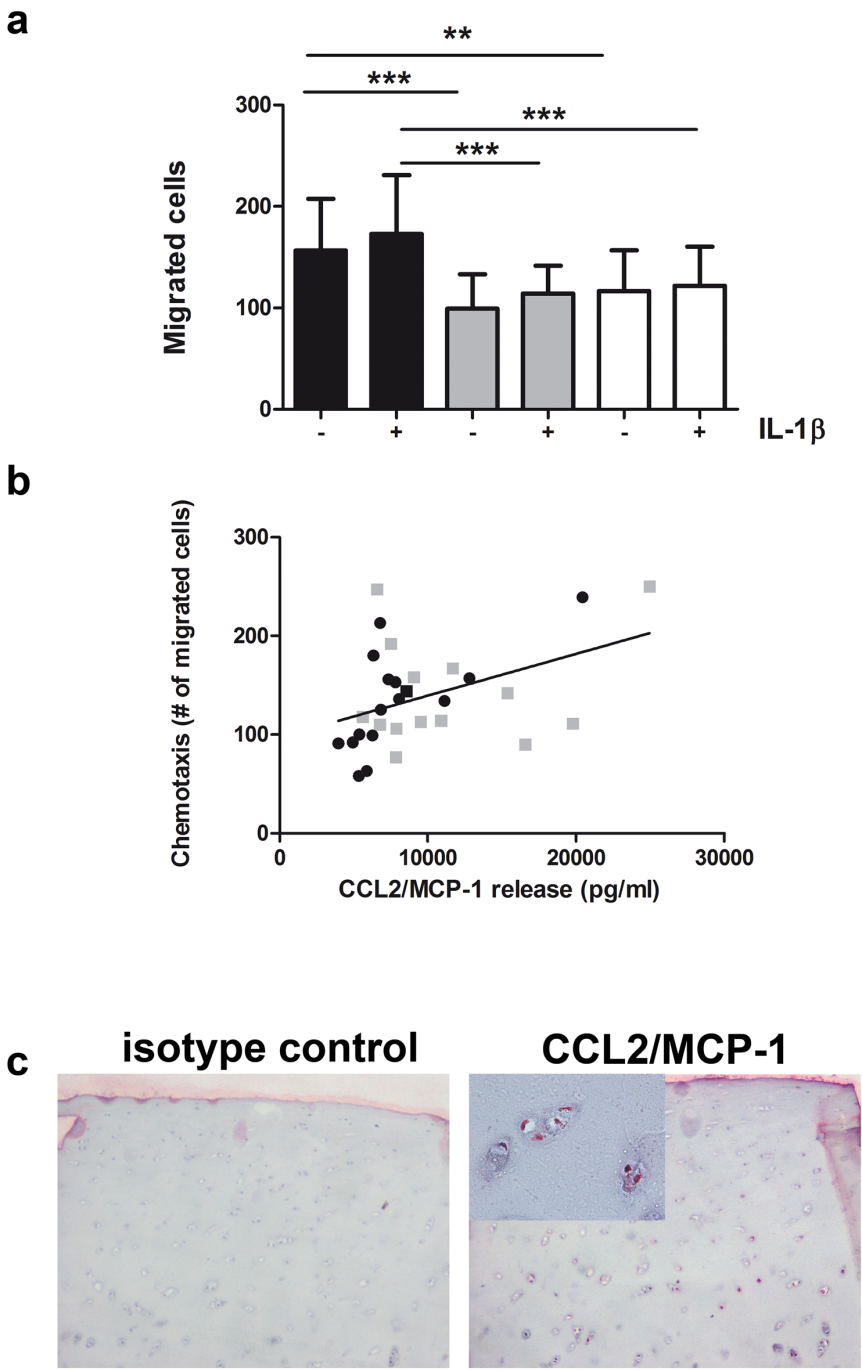
IKK dependency of monocyte recruitment following exposure to chondrocyte conditioned media. (**a**) Supernatants obtained from high density cultures (n = 7) of GL2 (black pattern), IKKαKD (gray pattern) or IKKβKD (white pattern) chondrocytes in control condition (−) or after IL-1β stimulation (24 h, +) were collected and assessed for the level of monocyte chemotaxis in Boyden Chambers. Data are reported as mean ± SD. The means of the groups were compared by ANOVA, followed by Tukey’s post hoc test. The differences were considered significant when p < 0.05 with *p < 0.05; **p < 0.01; and ***p < 0.001. (**b**) The extent of chemotaxis in term of number of migrated cells is highly correlated to CCL2/MCP-1 concentration (pg/ml), as evaluated by mean of the Pearson r (r = 0.428, p = 0.02, two-tailed, n = 30). Different symbols refers to basal (full black circles) or IL-1β stimulated samples (full gray boxes). (**c**) MCP-1 is expressed in osteoarthritic cartilage as assessed by immunohistochemistry (on the left isotype control; on the right cartilage section probed with anti-MCP-1 antibody, ×4 magnification and ×40 insert).

We collected supernatants from both monolayer (n = 3) and micromass cultures (n = 4) with and without IL-1β stimulation (24 h with 2 ng/ml IL-1β). Supernatants, along with control chemokines, were added to the lower wells of the chemotaxis chamber. Compared to control GL2 chondrocytes, supernatants collected from chondrocytes with either IKKα or IKKβ 80–90% penetrant KDs showed a strong inhibition of cell migration in both un-stimulated and IL-1β treated conditions (Fig. 3a).

IKKα or IKKβ KDs of 80–90% blunted the monocyte chemotactic potential of chondrocyte conditioned media. Under basal conditions CCL2/MCP-1 was the chemokine with the highest concentration in wild type chondrocyte conditioned media, and presented the strongest association with monocyte chemotaxis. Noteworthy, the chemotaxis data (expressed as the number of migrated monocytes) obtained with conditioned media from monolayer (2 sets from two independent patients corresponding to 12 samples) and micromass samples (2 sets from three independent patients corresponding to 18 samples significantly correlated with CCL2/MCP-1 protein release (r = 0.428, p = 0.02, n = 30) (Fig. 3b). The correlation was even stronger in the subset of monolayer samples, shown in Supplementary Fig. 3 (r = 0.94, p < 0.0001, n = 12). The correlation suggests that at least part of the dual IKK dependency for this inflammatory-like migration response was associated with the magnitude of CCL2/MCP-1 production. As chondrocyte conditioned media induced a strong migration response by primary monocytes under basal condition, this suggests a constitutive activation of ΙΚΚα and IKKβ in OA chondrocytes, likely reflecting the synovitis often complicating OA.

To confirm CCL2/MCP-1 involvement in OA pathophysiology, as already reported by Ni et al.^33^, we also found a clearly detectable protein expression in chondrocytes of the middle zone of OA cartilage (Fig. 3c). Noteworthy, this area, as well as the deep zone of articular cartilage derived from samples with advanced OA (as assessed with Safranin-O staining) also showed high expression of IKKα, otherwise not detectable in cartilage with conserved extracellular matrix (supplementary Fig. 6).

### Knockdown of either IKKα or IKKβ reduces IL-1β-induced NF-κB activation, with IKKαKD also impacting on chromatin remodeling

The differential involvement of the IKK NF-κB activating kinases downstream of IL-1β stimulation was assessed in western blots by kinetically comparing major signalling events in control (GL2), IKKα or IKKβKD chondrocytes cultured in monolayer.

**Figure 4:**
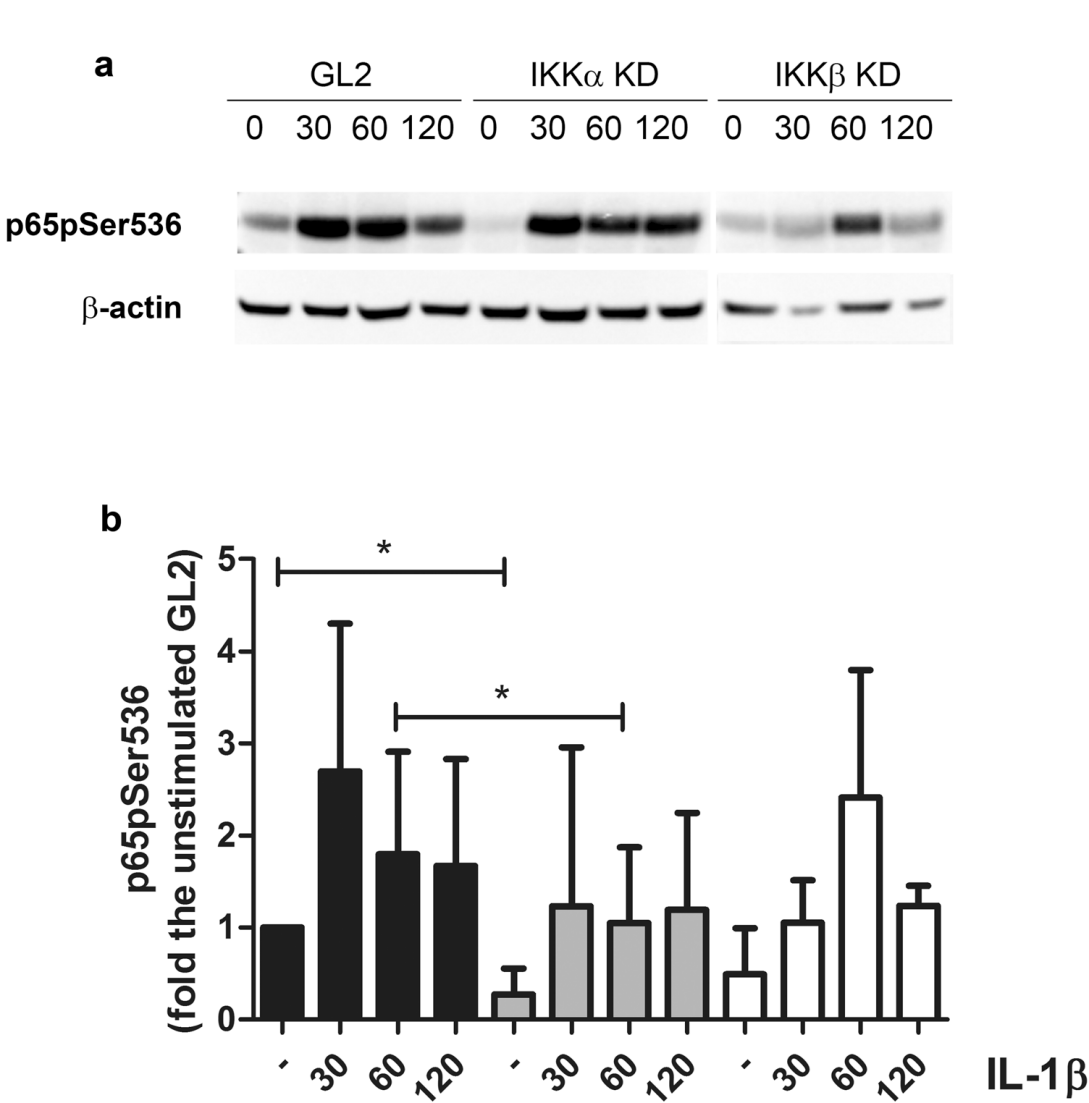
IKK dependency of p65phosphorylation. (**a**) A representative western blot (out of four performed) showing the kinetic of p65 phosphorylation (p65pSer536) in high density cultures of chondrocytes with either the control shRNA (GL2) or with IKKαKD or IKKβ KD. The cultures were either unstimulated (0) or treated with 2 ng/ml IL-1β for 30, 60 or 120 min. At the end of stimulation, the cells were recovered by trypsinization and counted. Total proteins derived from equal cell equivalents were run and blotted. β actin was used as a loading control. (**b**) Data obtained from the four experiments underwent densitometric analysis, and the cumulative results reported as mean ± SD fold increase the level of the control unstimulated GL2. The means of the groups were compared by the Student’s t test. The differences were considered significant when p < 0.05 with *p < 0.05. Different patterns are used for different phenotypes: GL2 black, IKKαKD gray, IKKβKD white. Both the basal and the 60 min p65pSer536 levels were higher in GL2 compared the IKKαKD level.

As shown in Fig. 4a (one representative western blot out of four performed) the levels of phosphorylated NF-κB p65 (RelA) were greatly enhanced as early as 30 min after IL-1β addition, in both GL2 control cells and IKKαKD. Compared to the GL2 control, in IKKαKD cells, a significantly reduced phosphorylation level was observed both in basal conditions and after 1 h of IL-1β stimulation (Fig. 4b showing the cumulative results) which corresponds to the initial phase of transcription according to previous findings^30^. As expected, the level of p65pSer536 was much lower in IKKβKD cells, and somehow delayed.

**Figure 5:**
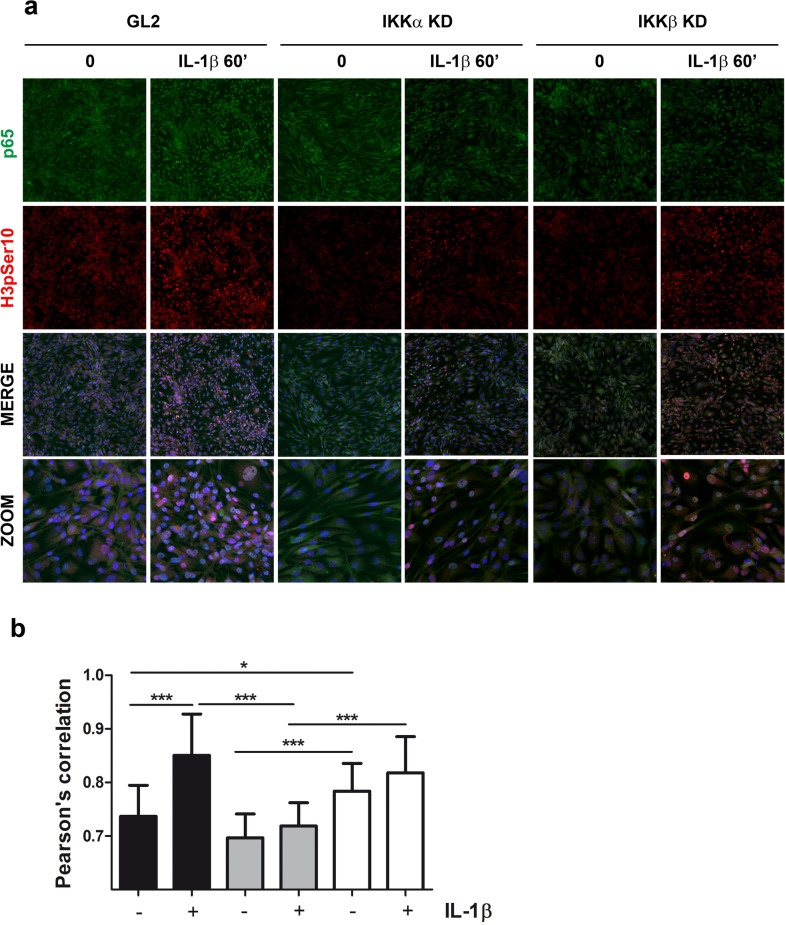
IKK dependency of co-localized p65 and H3pSer10. (**a**) Confocal analysis of the extent of co-localization of p65 signal (green) with that corresponding to the phosphorylated Serine 10 of the histone H3 (red) in both basal conditions and after 60 min stimulation with 2 ng/ml IL-1β. The distinct signals are shown, together with the merged images including Hoechst 33342 as a nuclear counterstaining and a ×3 zoom in order to better appreciate the co-localized signal at the nuclear level, appearing as pink dots. (**b**) Analysis of the extent of co-localization derived from 26 different images for each condition, spread in the whole samples. Data are reported as mean ± SD. The means of the groups were compared by ANOVA, followed by Tukey’s post hoc test. The differences were considered significant when p < 0.05 with *p < 0.05; **p < 0.01; and ***p < 0.001. In both IKK KD, the IL-1β dependent induction is greatly blunted compared to the IKK proficient GL2 chondrocytes. Noteworthy, co-localized signals were significantly lower in IKKαKD compared to IKKβKD in both basal conditions and after IL-1β stimulation. IKKαKD data after IL-1β were significantly lower compared to both GL2 and IKKβKD samples.

Exploiting immunofluorescence and confocal microscopy analysis, as shown in Fig. 5 we also investigated the extent of the combined occurrence of nuclear localized p65 (green signal in the nuclei: activated NF-κB monomer) and H3pSer10 (red signal for histone modification occurring after inflammatory cytokine delivery, which indicates IKKα epigenetic activity required for transcription initiation). The analysis was carried out at 1 h post IL-1β addition. Figure 5a shows representative fields out of 36–45 analyzed for each condition. The two signals were already evident at basal level, but after IL-1β stimulation their levels were enhanced in the GL2 control condition. Nuclear p65 was diminished in both IKKα and IKKβ KD cells, while H3pSer10 was more markedly reduced in IKKαKD cells, as expected. The combined assessment of the two signals is evident as a pink pattern designating the nuclear shape. A more analytical evaluation of the co-localization, assessed by means of ANOVA with Tukey post-hoc correction, is shown in Fig. 5b. The analysis confirmed an IL-1β dependent significant increase in GL2 samples compared to their un-stimulated counterparts, but not in each of the IKK KD samples. Noteworthy, the co-localization levels in IL-1β IKKαKD samples were statistically lower compared to both IL-1β GL2 and IL-1β IKKβKD. IKKβKD had higher un-stimulated levels compared to both GL2 and IKKαKD un-stimulated samples.

## Discussion

Our findings indicate that both CCL2/MCP-1 level and monocyte chemotaxis are significantly higher in IKK proficient cells, with a mild increase after IL-1β stimulation. The latter might be due to the status of pre-activated NF-κB in high density and differentiated chondrocytes, as also previously reported^25^. Despite a clear IL-1β dependent RNA increase (8 h) for most chemokine tested, protein levels at the 24 h time point indicate no significant changes, suggesting the existence of epigenetic post-transcriptional controls possibly intervening after the first wave of mRNA transcription^34^.

These findings are intriguing in the perspective of developing strategies to target synovitis, which is responsible for pain and swelling, and the positive feedback loops that amplify joint damage^5^.

At its onset, OA is primarily a disease of articular cartilage, prompted by an array of different risk factors and etiologies that trigger shared signalling mechanisms towards a shared outcome of a failure in homeostatic mechanisms^35^ thus favouring catabolism sustained by activation of matrix-degrading proteinases^36^. Early after onset, crosstalk among the joint tissues occurs so that established OA is now known to be a complex condition affecting the whole joint where all joint compartments contribute to disease progression^5^. In particular, OA is often associated with low-grade synovitis^37–39^. Moreover, recruitment of monocytes to the synovium has been evidenced as a pivotal event in driving synovial inflammation and linking innate immunity to OA^18,19^. Peripheral blood monocytes are recruited to the synovium by chemokines that derive from either chondrocytes or synovial fibroblasts^40^. In the DMM OA model, CCL2 is among the earliest induced genes, in a matter of hours after surgery^20^, and highly involved in pain rather than in cartilage degradation. This is in agreement with recent studies pointing to a central role of the CCL2/CCR2 axis at the level of the dorsal root ganglia in the onset of pain hypersensitivity^41^.

Further studies confirmed the pivotal role of CCL2/MCP-1 in mediating monocyte recruitment and their activation into macrophages, inflammation and cartilage destruction in both the DMM OA model and in human OA. These effects were significantly delayed in the surgical OA DMM model by selective targeting of the CCL2/CCR2 axis^42^.

In keeping with this, a recent meta-analysis pointed to CCL2/MCP-1 as a potential biomarker for diagnosis and progression of human OA^33^. Other studies have shown that CCL2/MCP-1 serum levels are reliable markers of OA “severity” both for structural degradation^43^ and symptoms^44^. Interestingly, the latter study included CCL2/MCP-1 in a subset of six biomarkers that are also highly correlated with synovial biomarkers of macrophage activation and are helpful to identify an inflammatory OA endotype with high risk of progression. Therefore, CCL2/MCP-1 is a convenient target whose tuning can significantly affect the quality of patient life.

CCL2/MCP-1 is a genuine target of NF-κB^45^, a signalling pathway that functions as a central hub for many chronic inflammatory diseases such as OA^8^. A large amount of evidence supports a pivotal role of NF-κB in OA pathogenesis in both initiation and progression^9^. Several attempts have been made to counteract OA via targeting NF-κB and/or its activating kinases^46^. As also recently reviewed^47^, the current state of the art indicates some redundancy for the two IKKs in canonical NF-κB activation and inflammatory responses^48–51^. Some cell specificities have been reported, but in mouse embryonic fibroblasts two different functional genomics studies with stably silenced IKKα or IKKβ pointed at their shared and cooperating role in canonical NF-κB activation. This was firstly reported by^51^ and confirmed by defects in main signalling events (IκBα and p65 phosphorylation^50^) or in transcriptional regulation of canonical NF-κB target genes^52^, whose largest subset were found to be co-dependent on both IKKα and IKKβ. Interestingly, the latter paper indicated that the signalosome IKKs are required to keep a basal level of NF-κB activation in the absence of extracellular stimuli. Our array and real Time PCR results suggest that IKKα has a prevalent role in sustaining CCL2/MCP-1 transcription, given its lowest basal levels in IKKαKD compared to both GL2 and IKKβKD chondrocytes. Interestingly, both basal and IL-1β induced levels of CCL2/MCP-1 in IKKβKD chondrocytes were higher compared to IKKαKD chondrocytes, possibly due to the already reported enhanced release of IL-1β associated with IKKβ inhibition^53^.

On the other hand, the two IKKs exert different roles in morphogenesis in keeping with different substrates and different non-NF-κB dependent roles^46^.

Another notable peculiar role of IKKα is functioning as a “nucleosomal kinase” exerting an activity in chromatin remodelling after cytokine induction, namely the ability to phosphorylate serine 10 in histone H3 (H3pSer10). The phosphorylation of H3pSer10 in conjunction with its subsequent acetylation, opens the chromatin to activate transcription of NF-κB responsive genes^12,13^ also favoured by an IKKα mediated de-repression of SMRT^54^. This is particularly critical for a subset of genes which includes CCL2/MCP-1^30^, whose promoters are thus prompted for transcription initiation by IKKα via H3pSer10. Therefore, it is conceivable that the spatial concurrence of H3pSer10 and p65 being localized in the nucleus is highly suggestive of the extent of NF-κB dependent transcription, in particular for CCL2/MCP-1. It has been shown that H3pSer10 is also dependent on p38MAPK activity, increased in high density chondrocytes^25^, but IKKα is the kinase that mediates its induction downstream of the delivery of inflammatory cytokines^12,13,55^. H3pSer10 induction following inflammatory stimuli has been shown to be required for a subset of inflammatory factors of high relevance in OA: CCL2/MCP-1, IL-6 and CXCL8/IL-8^30^ as confirmed by CHIP via anti- H3pSer10 antibody. The IKKα > H3pSer10 epigenetic event is also involved in transcription of CCL5/RANTES and other NF-κB target genes (MnSOD, IκBα and Cox-2) as demonstrated by CHIP via an anti-IKKα antibody^56^. Interestingly, comparison of CHIP assays performed with anti-IKKα or anti-RelA antibodies showed different kinetics of binding to the promoters. However, at 1 h (the time we selected for the colocalization analysis) both IKKα and RelA were bound to the promoters. In keeping with this information, our data show that IKKαKD abrogated IL-1β dependent increased CCL5/RANTES transcription in chondrocytes.

Interestingly, our analysis of H3pSer10 and p65 co-localized signals indicates that silencing of either IKK might abolish their increase after 1 h of IL-1β stimulation. Collectively our results suggest that IKKαKD acts mostly by suppressing H3pSer10 signals while IKKβKD by reducing the levels of p65pSer536. In addition, Gloire et al. further reported that IKKαKD prevented p65 binding to the CCL2/MCP-1 promoter^57^.

Summarizing the above described evidence, the current view is that IKKβ is the kinase “mainly” involved in canonical NF-κB activation, while IKKα is the only kinase involved in delayed, non canonical NF-κB activation^9^. However, canonical NF-κB activation also relies on the involvement of IKKα^51,52^, that cooperates with IKKβ in phosphorylating IκBα and p65/RelA and, in addition, plays a unique role in chromatin remodeling independent of NF-κB required to start CCL2/MCP-1 transcription^12^. Therefore, both IKKs can be targeted to reduce CCL2/MCP-1 and to counteract its IL-1β dependent induction and monocyte recruitment and activation.

Functional genomic studies have previously shown that IKKβ deletion has the same phenotype of blocking NF-κB activation with a dominant negative IκBα mutant, which reduces the pro-survival pathways that spare cells from apoptosis, thus representing an attractive therapeutic choice for cancer^58^, but not for the treatment of chronic diseases with an inflammatory background such as OA. This is also in line with our previous findings obtained in 3-D chondrocyte cultures, a culture model which recapitulates differentiation progression from hypertrophy to terminal differentiation. In this culture model only IKKαKD exhibits protection from cell death^15^.

Furthermore, critical factors might be considered in the choice of targeting IKKs as the level of their expression and the relevance of their role in the tissue of interest. Indeed, we found that IKKα expression is almost switched off in normal healthy cartilage^59^, that must be kept in a defined post-mitotic differentiation window, while its re-expression in OA cartilage is able to drive the differentiation program towards hypertrophy and terminal differentiation^16^ as also reported for skin by functional genomics^60,61^. Therefore, IKKα may represent a dispensable protein in normal healthy cartilage, in keeping with its being a target gene of NOTCH^62^, that drives transition to hypertrophy and terminal differentiation^63^. The latter processes have been considered as developmental models for OA pathogenesis^14,64^. A significantly increased IKKα expression in cartilage was observed in the DMM model in the stage of early OA^65^. Noteworthy, as we previously reported a meta-analysis of the transcriptome of WT vs IKKαKD chondrocytes with that of normal vs late OA cartilage^66^, indicated that most gene probe sets differentially expressed in GL2 vs IKKαKD micromasses (315/436 = 72%; p value < 0.02) were also differentially expressed in human late OA vs. normal cartilage^59^, and IKKα was among these genes^59^. IKKα’s role in differentiation has been confirmed as NF-κB and kinase independent, both in skin^67^ and in cartilage differentiation^16^.

Therefore, IKKα might represent a convenient target to tackle at the joint level, to significantly reduce inflammation and innate immunity activation in OA.

In keeping with the above statement, some recently published findings indicate attenuation of extracellular matrix remodelling and OA progression as assessed by the OARSI score in in vivo OA models with pharmacological^65^ or genetic^68^ IKKα inhibition. Noteworthy, in the latter report dealing with a DMM model carried out prior to skeletal maturity^69^, conditional knockout of IKKα hampered the proper organization of the growth plate, and was responsible for a higher level of apoptosis of growth plate chondrocytes. This further discloses functional differences between articular (permanent) and growth plate (transient) cartilage. In growth plate, chondrogenesis (the process that leads to the formation of the cartilage anlagen starting from mesenchymal precursors) is followed by hypertrophy and terminal differentiation^70^. The latter processes, that must be prevented in healthy cartilage but that are improperly triggered in OA, are also driven by re-expression of NOTCH-1^63^. It is conceivable that the lack of IKKα favours apoptosis in light of the anti-apoptotic role of NOTCH-1–IKKα interaction^71^.

A local, “articular” IKKα tuning with the purpose of reducing monocyte activation and synovium inflammation may also be obtained via intra-articular injection of N-acetyl phenylalanine glucosamine (NAPA)^65^, a glucosamine derivative able to reduce IKKα nuclear translocation and phosphorylating activity on Histone H3 thereby effectively reducing CCL2/MCP-1^27,72^ or via nanomedicine approaches with delivery of nanoparticles coated with target specific siRNA, a novel approach already tested in vivo as a promising therapeutic avenue for Rheumatoid Arthritis^73^.

## Supplementary Information

Supplementary Information.

## Data Availability

Data reported in the study are available upon request to the corresponding author.
