# Supplementary material for: Basal and IL-1β enhanced chondrocyte chemotactic activity on monocytes are co-dependent on both IKKα and IKKβ NF-κB activating kinases
Source: Sci Rep. 2021 Nov 4;11:21697. doi: 10.1038/s41598-021-01063-2 (PMC8568921; doi:10.1038/s41598-021-01063-2)
Supplement: Supplementary file 1 — Supplementary Information. [file 41598_2021_1063_MOESM1_ESM.pdf]

SUPPLEMENTARY MATERIAL

Basal and IL-1β enhanced chondrocyte chemotactic activity on monocytes are co-dependent on both IKKα and IKKβ NF-κB activating kinases

Eleonora Olivotto[1], Manuela Minguzzi[2], Stefania D'Adamo[2], Annalisa Astolfi[3], Spartaco Santi[4,5], Mariagrazia Uguccioni[6,7], Kenneth B. Marcu[8#] and Rosa Maria Borzì[9#]

[1]Laboratorio RAMSES, IRCCS Istituto Ortopedico Rizzoli, Bologna, Italy

[2]Dipartimento di Scienze Mediche e Chirurgiche (DIMEC), Università di Bologna, Bologna, Italy

[3]Department of Translational Medicine, University of Ferrara, Ferrara, Italy

[4]CNR Institute of Molecular Genetics "Luigi Luca Cavalli-Sforza", Unit of Bologna, Bologna, Italy.

[5]IRCCS, Istituto Ortopedico Rizzoli, Bologna, Italy

[6]Institute for Research in Biomedicine, Università della Svizzera italiana, Bellinzona, Switzerland

[7]Dept. of Biomedical Sciences, Humanitas University, Pieve Emanuele – Milan, Italy

[8]Departments of Biochemistry and Cell Biology and Pathology, SUNY, Stony Brook, NY, United States

[9]Laboratorio di Immunoreumatologia e Rigenerazione Tissutale, IRCCS Istituto Ortopedico Rizzoli, Bologna, Italy

Correspondence should be addressed to Rosa Maria Borzì; rosamaria.borzi@ior.it

# = equal contributors

S1

a

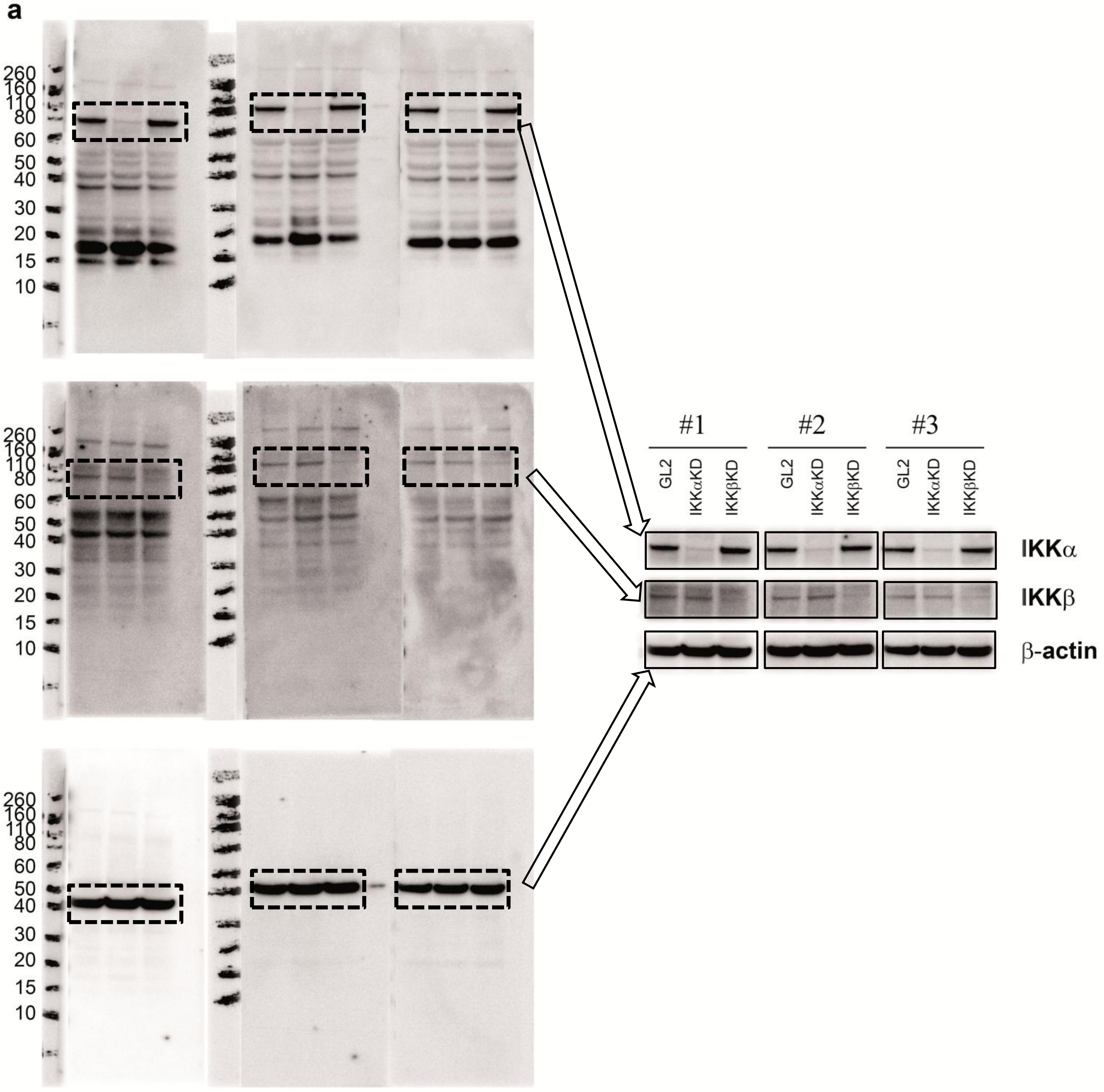
260
160
110
80
60
50
40
30
20
15
10
#1
#2
#3
GL2
IKKαKD
IKKβKD
IKKα
IKKβ
β-actin

b

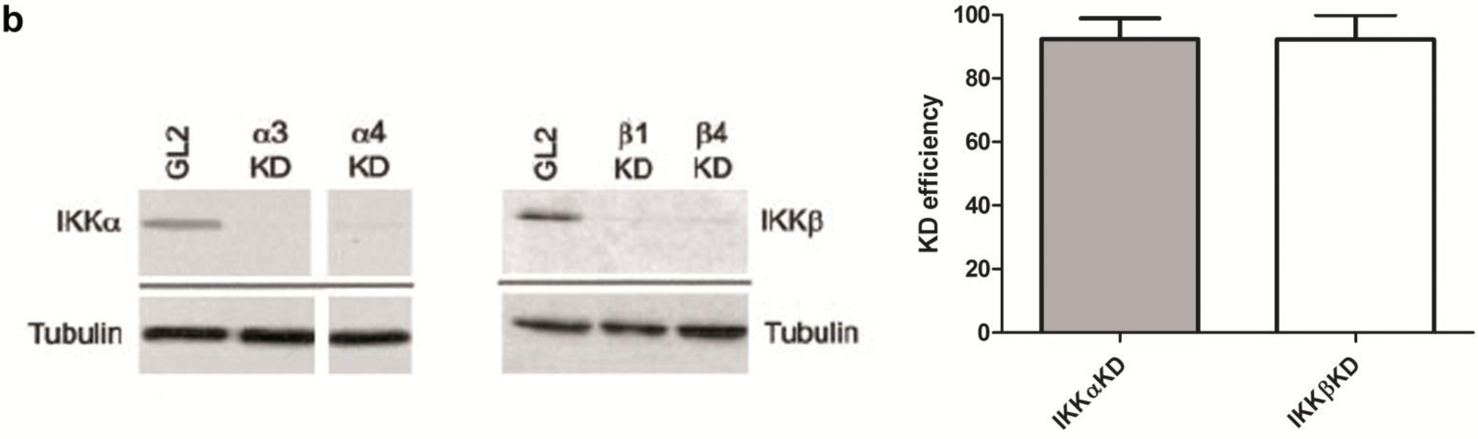
GL2
α3 KD
α4 KD
IKKα
Tubulin
GL2
β1 KD
β4 KD
IKKβ
Tubulin
KD efficiency
0
20
40
60
80
100
IKKαKD
IKKβKD

S2

Array Layout

RPS27A<br>1	AGTRL1<br>2	BDNF<br>3	BLR1<br>4	C20orf175<br>5	C5<br>6	CCBP2<br>7	CCL1/i-309<br>8
CCL11_Eotaxin<br>9	CCL13-MCP-4<br>10	CCL15/HCC-2<br>11	CCL16/HCC-4<br>12	CCL17/TARC<br>13	CCL18/PARC<br>14	CCL19/ELC<br>15	CCL2/MCP-1<br>16
CCL20/MIP-3α<br>17	CCL21/SLC<br>18	CCL23/MPIF-1<br>19	CCL24/Eotaxin-2<br>20	CCL25/TECK<br>21	CCL26/Eotaxin-3<br>22	CCL27/CTACK<br>23	CCL28<br>24
CCL3/MIP-1α<br>25	CCL4/MIP-1β<br>26	CCL4L1<br>27	CCL5/RANTES<br>28	CCL7/MCP-3<br>29	CCL8/MCP-2<br>30	*CCR1*<br>31	*CCR2*<br>32
CCR3<br>33	*CCR4*<br>34	*CCR5*<br>35	*CCR6*<br>36	*CCR7*<br>37	*CCR8*<br>38	*CCR9*<br>39	CCRL1<br>40
CCRL2<br>41	CKLF<br>42	CMTM1<br>43	CMTM2<br>44	CMTM3<br>45	CMTM4<br>46	CMTM5<br>47	CMTM6<br>48
CMTM7<br>49	CMTM8<br>50	CMKLR1<br>51	CMKOR1<br>52	CSF3<br>53	CX3CL1<br>54	CX3CR1<br>55	CXCL1/GROα<br>56
CXCL10/IP-10<br>57	CXCL11/I-TAC<br>58	CXCL12/SDF-1α/β<br>59	CXCL13/BCA-1<br>60	CXCL14/BRAK<br>61	CXCL16/?<br>62	CXCL2/GROβ<br>63	CXCL3/GROγ<br>64
CXCL5/ENA-78<br>65	CXCL6/GCP-2<br>66	CXCL9/Mig<br>67	*CXCR3*<br>68	*CXCR4*<br>69	*CXCR6*<br>70	CYFIP2<br>71	ECGF1<br>72
EPO<br>73	FY<br>74	GDF5<br>75	GPR109B<br>76	*CCR10*<br>77	GPR31<br>78	C5R1<br>79	GPR81<br>80
HIF1A<br>81	IL13<br>82	IL16<br>83	IL18<br>84	IL1A<br>85	IL4<br>86	IL8<br>87	*IL8RA*<br>88
IL8RB<br>89	LTB4R<br>90	MMP2<br>91	MMP7<br>92	MYD88<br>93	NFKB1<br>94	PF4<br>95	PPBP<br>96
PRL<br>97	RGS13<br>98	RGS3<br>99	SCYE1<br>100	SDF2<br>101	SLIT2<br>102	TCP10<br>103	TLR2<br>104
TLR4<br>105	TNF<br>106	TNFRSF1A<br>107	TNFSF14<br>108	TREM1<br>109	TREM2<br>110	VHL<br>111	XCL1<br>112
XCR1<br>113	Blank<br>114	PUC18<br>115	Blank<br>116	Blank<br>117	AS1R2<br>118	AS1R1<br>119	AS1<br>120
GAPDH<br>121	B2M<br>122	HSPCB<br>123	HSPCB<br>124	ACTB<br>125	ACTB<br>126	BAS2C<br>127	BAS2C<br>128

S3

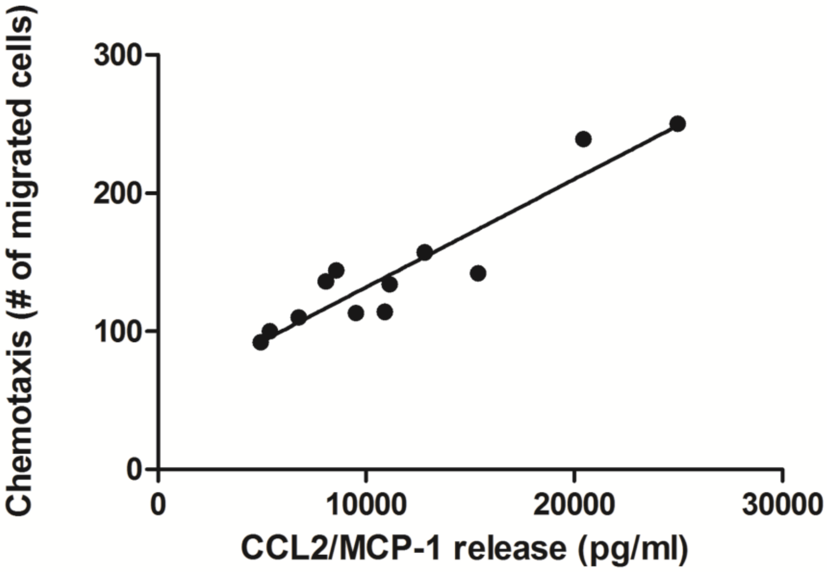

Chemotaxis (# of migrated cells)
300
200
100
0
0
10000
20000
30000
CCL2/MCP-1 release (pg/ml)

S4

a

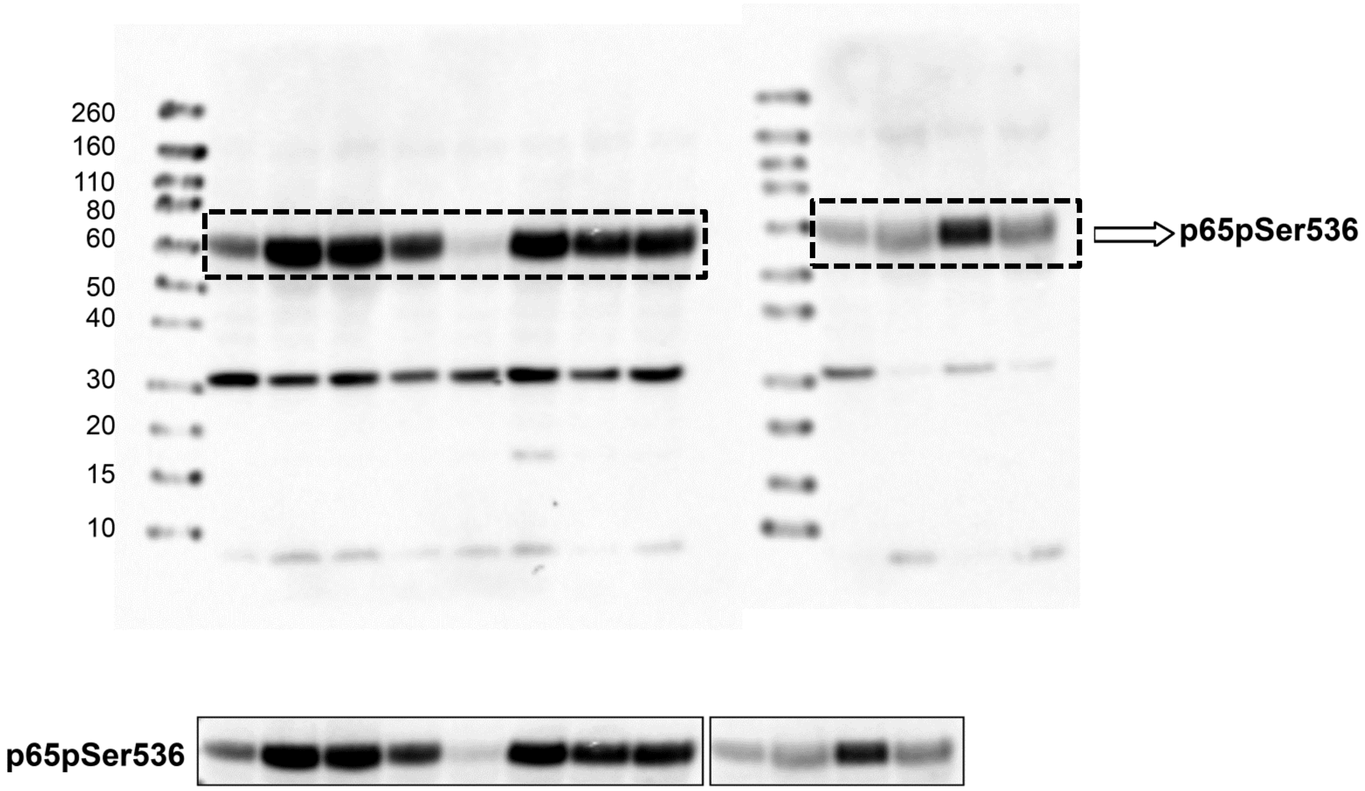
260
160
110
80
60
50
40
30
20
15
10
p65pSer536
p65pSer536

b

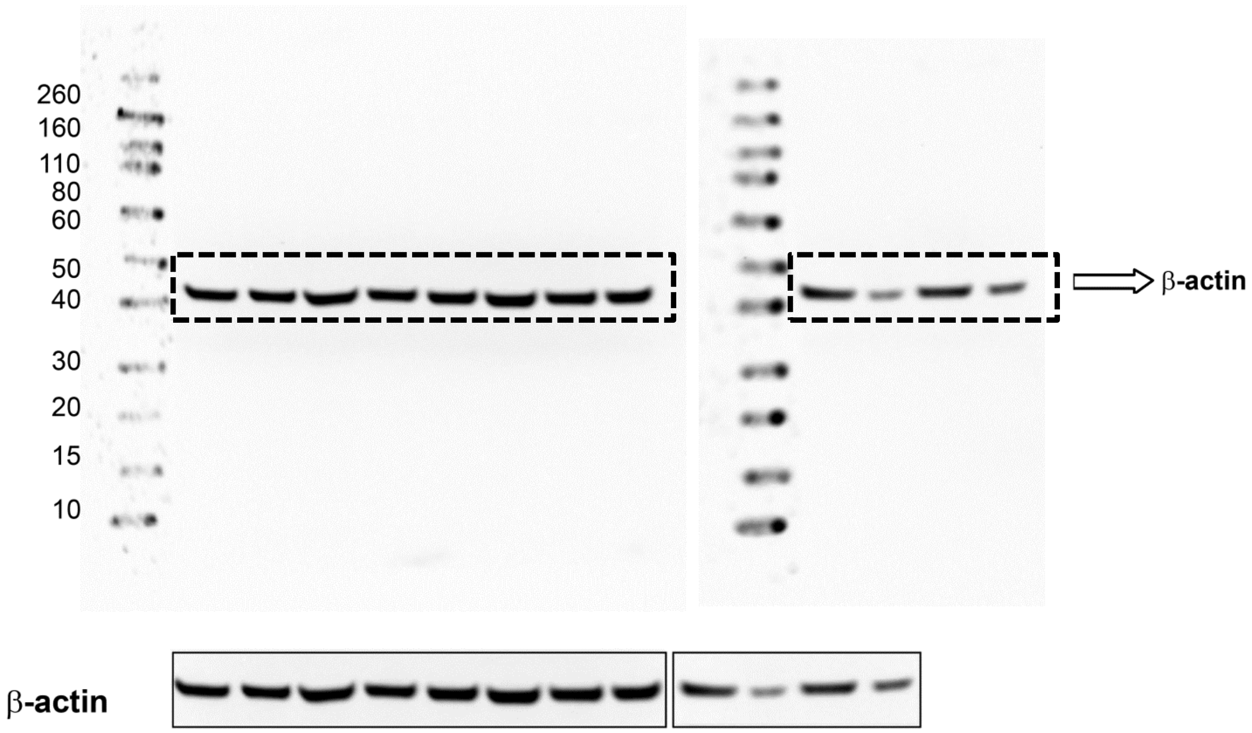
260
160
110
80
60
50
40
30
20
15
10
β-actin
β-actin

S5

a

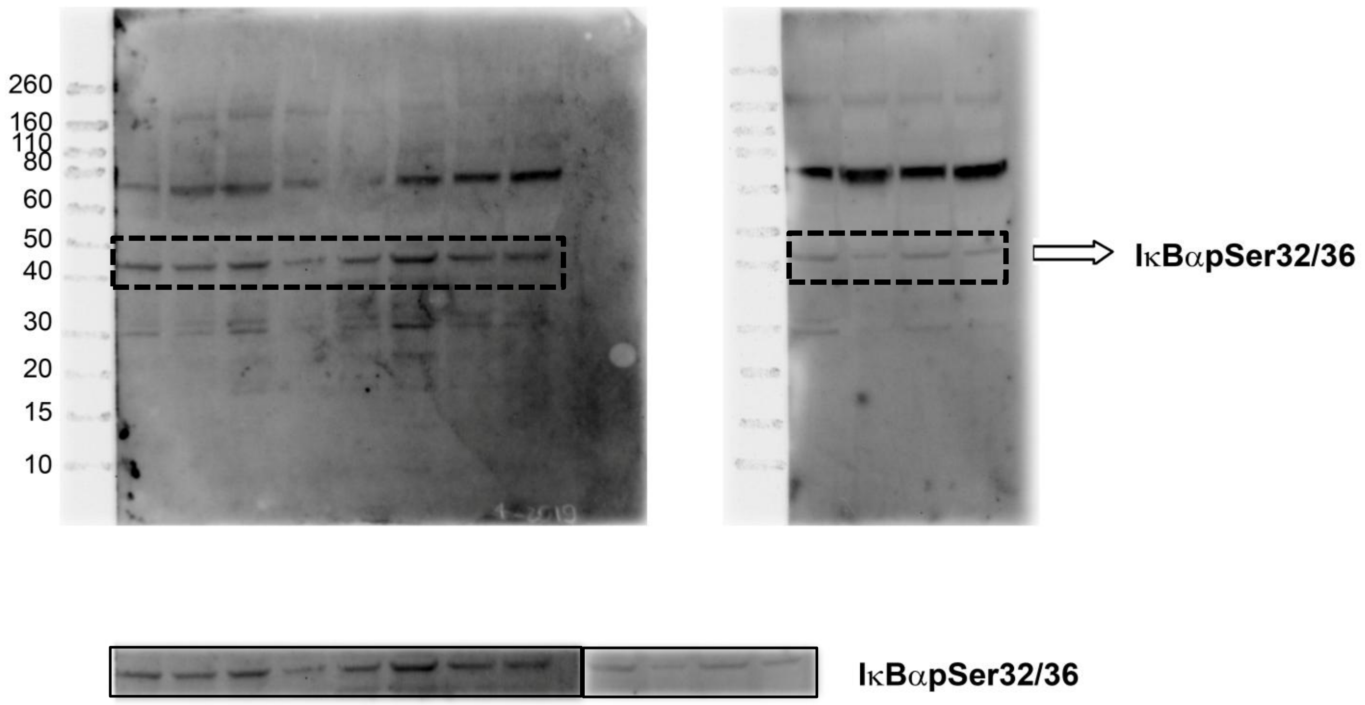

260
160
110
80
60
50
40
30
20
15
10
IκBαpSer32/36
IκBαpSer32/36

b

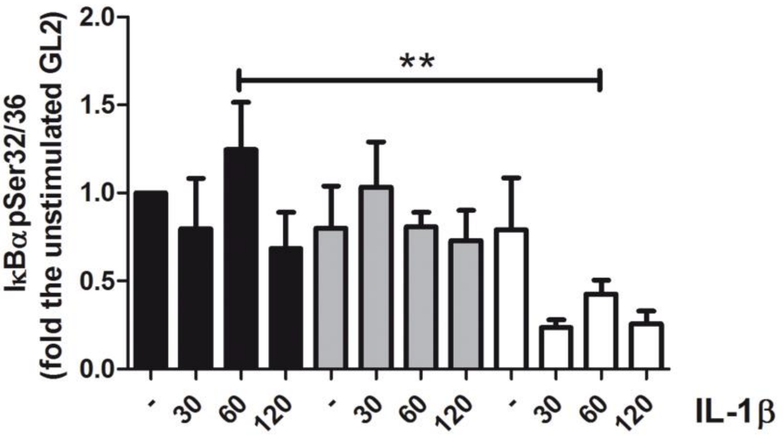

IκBαpSer32/36
(fold the unstimulated GL2)
2.0
1.5
1.0
0.5
0.0
**
-
30
60
120
-
30
60
120
-
30
60
120
IL-1β

S6

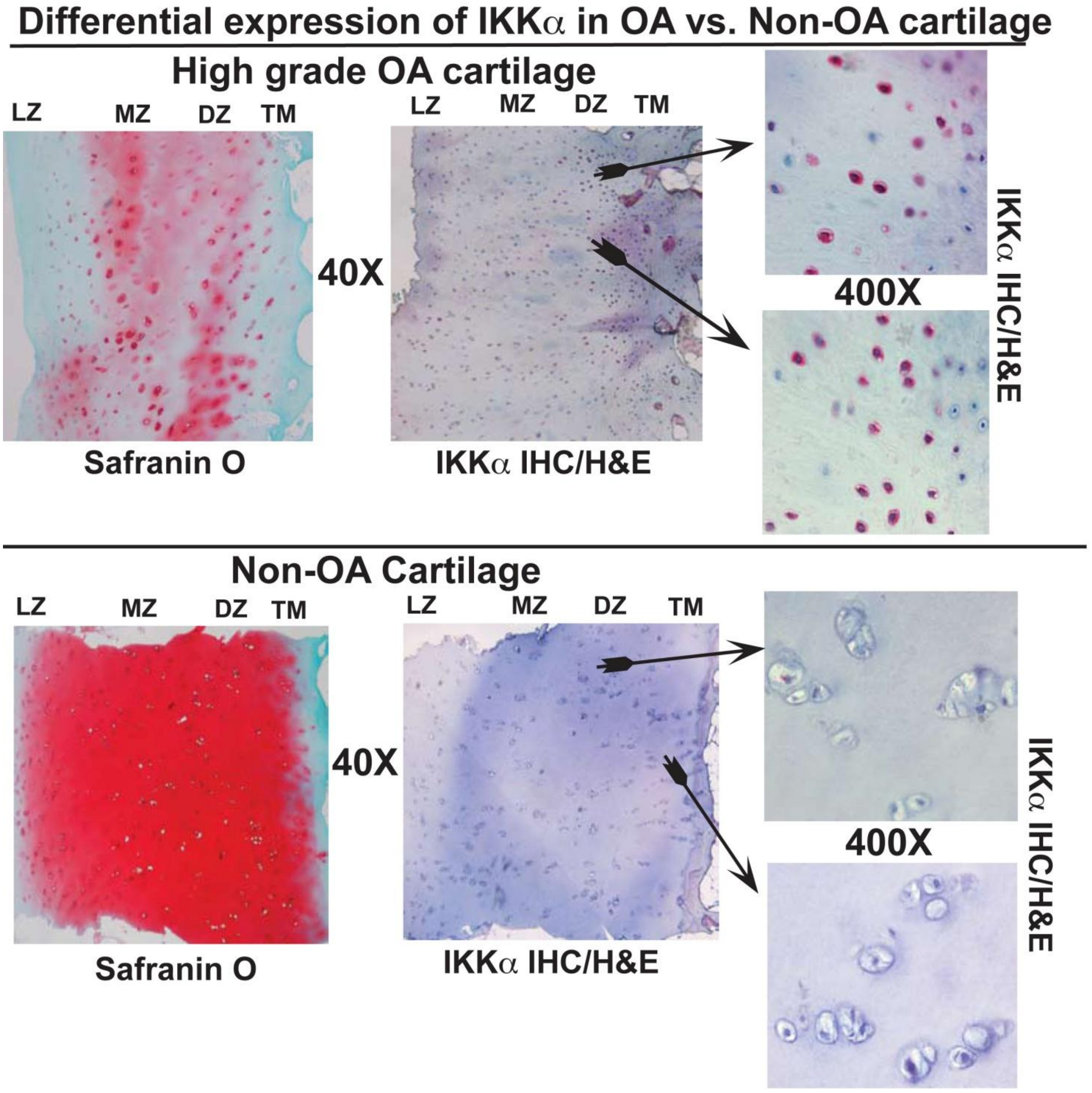
Differential expression of IKKα in OA vs. Non-OA cartilage
High grade OA cartilage
LZ
MZ
DZ
TM
40X
Safranin O
LZ
MZ
DZ
TM
IKKα IHC/H&E
400X
IKKα IHC/H&E
Non-OA Cartilage
LZ
MZ
DZ
TM
40X
Safranin O
LZ
MZ
DZ
TM
IKKα IHC/H&E
400X
IKKα IHC/H&E

S7

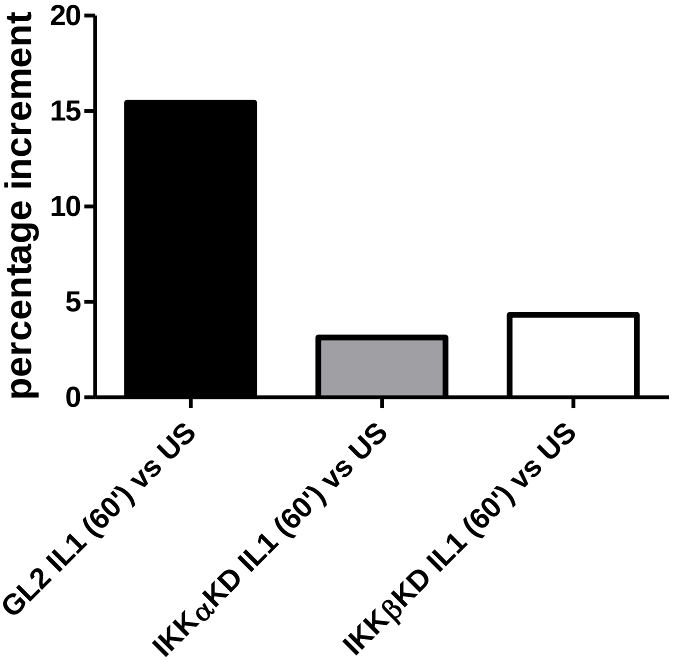

percentage increment
20
15
10
5
0
GL2 IL1 (60') vs US
IKKαKD IL1 (60') vs US
IKKβKD IL1 (60') vs US

Legend to Supplementary Figures

Figure S1: a) Representative examples obtained with three different primary cultures treated with retroviral transduction of shRNA to obtain GL2 control chondrocytes, IKKαKD and IKKβKD chondrocytes. Full blots (left) obtained with the same membranes probed with anti–IKKα, anti-IKKβ and anti-β-actin antibodies. At the end of puromycin selection, the cells were recovered by trypsinization and counted. Total proteins derived from equal cell equivalents were loaded in NuPAGE Novex 4-12% Bis-Tris gels. Samples were run with MES buffer, along with Novex Sharp Pre-Stained Protein Standards. After protein transfer, the lanes containing the standards were cut from the membranes containing the samples. To assess the molecular weight of western blot stained bands the pre-stained bands of the markers were highlighted by mean of a Glow Writer pen (http://divbio.com/glow-writerpen.aspx) and at the end of western blotting, the lanes containing the Protein Standards were juxtaposed to the original membranes. The dashed rectangles indicates the bands included in the crops, and on the right the arrows indicates the protein bands used to set up the right cumulative figure. **b)** left: example taken from our first publication (Olivotto, E. *et al. Arthritis Rheum* **58**, 227-239, doi:10.1002/art.23211 (2008)) of the efficiency of different vectors to obtain knockdown of either IKKα or IKKβ. Cumulative evaluation of KD efficiency (mean ± SD) for either IKKα and IKKβKD cultures. Data derived from 10 different infections.

Figure S2: Layout of the probes of the Oligo GEArray microarray human chemokines and receptors microarray (OHS-022, SuperArray), used for Figure 1a and 1b of the main manuscript.

Figure S3: The extent of chemotaxis in term of number of migrated cells is highly correlated to the CCL2/MCP-1 concentration (pg/ml), as evaluated by mean of the Pearson r ($r=0.94$, $p<0.0001$, two-tailed, $n=12$). The figure reports the correlation between chemotaxis and CCL2/MCP-1 concentration obtained from unstimulated and IL-1β stimulated samples (GL2, IKKαKD and IKKβ KD) derived from primary chondrocyte cultures established from two different patients. The figure represents the subset of the data obtained with high density monolayer cultures.

Figure S4: Full blots used to derive the p65pSer536 **(a)** and β-actin **(b)** results shown in Figure 4A of the main manuscript obtained with lysates of high density cultures of chondrocytes with either the control shRNA (GL2) or with IKKαKD or IKKβKD. At the end of stimulation, the cells were recovered by trypsinization and counted. Total proteins derived from equal cell equivalents were loaded in NuPAGE Novex 4-12% Bis-Tris gels. Samples were run with MES buffer, along with

Novex Sharp Pre-Stained Protein Standards. After protein transfer, the lanes containing the standards were cut from the membranes containing the samples. To assess the molecular weight of western blot stained bands the pre-stained bands of the markers were highlighted by mean of a Glow Writer pen (http://divbio.com/glow-writerpen.aspx) and at the end of western blotting, the lanes containing the Protein Standards were juxtaposed to the original membranes. The dashed rectangles indicates the bands included in the crops, and on the right the arrow indicates the protein bands used to set up Figure 4a, whose cumulative image is reported below each set of full blots.

Figure S5: **a)** The same blots used to assemble Figure 4 were used to assess the level of IκBαpSer32/36. The figure shows the results obtained with the same membrane shown in Figure S4, therefore the housekeeping loading control is the same shown in Figure S4. **b)** Data obtained from the three experiments underwent densitometric analysis, and the cumulative results reported as mean ± SD fold increase the level of the control unstimulated GL2. The means of the groups were compared by the Student's t test. The differences were considered significant when $p < 0.05$ with *$p < 0.05$; **$p < 0.01$; and ***$p < 0.001$. Different patterns are used for different phenotypes: GL2 black, IKKαKD gray, IKKβKD white. The 30 min IκBαpSer32/36 levels were higher in GL2 compared to the levels in the IKKβKD level.

Figure S6: IKKα expression was analyzed by immunohistochemistry (IHC), with hematoxylin-eosin, (H&E) as a nuclear counterstaining in sections of full thickness explants of cartilage and subchondral bone derived from areas with conserved (Non-OA) versus areas with marked perturbation of cartilage extracellular matrix (OA), as assessed by Safranin-O staining. As indicated, magnification was 40x for full depth images, while details shown on the right were taken at 400x magnification. LZ= lining zone; MZ= middle zone; DZ=deep zone; TM=tidemark. Images were obtained from cartilage slides processed for IHC as detailed in Materials and Methods of the main manuscript. Primary antibody was the Pharmingen mouse monoclonal anti-IKKα used at 5µg/ml and compared with the isotype control (Dako IgG2b at the same concentration).

Figure S7: As shown in Figure 5 of the main manuscript, immunofluorescence and confocal microscopy analysis, were used to investigated the extent of the combined occurrence of nuclear localized p65 (green signal, activated NF-κB monomer) and H3pSer10 (red signal for histone modification occurring after inflammatory cytokine delivery, which indicates IKKα epigenetic activity required for transcription initiation). The analysis was carried out at 1 hour post IL-1β addition. The graph summarizes the mean IL-1β dependent percentage increment of the signal compared to the basal level for each condition.
